# Ultralow flow liquid chromatography and related approaches: A focus on recent bioanalytical applications

**DOI:** 10.1002/jssc.202300440

**Published:** 2023-08-01

**Authors:** Michal Greguš, Alexander R. Ivanov, Steven Ray Wilson

**Affiliations:** 1 Department of Chemistry and Chemical Biology, Barnett Institute of Chemical and Biological Analysis, Northeastern University, Boston, Massachusetts, USA; 2 Hybrid Technology Hub-Centre of Excellence, Institute of Basic Medical Sciences, University of Oslo, Oslo, Norway; 3 Department of Chemistry, University of Oslo, Oslo, Norway

**Keywords:** Bioanalysis, Monolith, Omics, Open tubular LC, Ultralow flow LC

## Abstract

Ultralow flow LC employs ultra-narrow bore columns and mid-range pL/min to low nL/min flow rates (i.e., ≤20 nL/min). The separation columns that are used under these conditions are typically 2–30 μm in inner diameter. Ultralow flow LC systems allow for exceptionally high sensitivity and frequently high resolution. There has been an increasing interest in the analysis of scarce biological samples, for example, circulating tumor cells, extracellular vesicles, organelles, and single cells, and ultralow flow LC was efficiently applied to such samples. Hence, advances towards dedicated ultralow flow LC instrumentation, technical approaches, and higher throughput (e.g., tens-to-hundreds of single cells analyzed per day) were recently made. Here, we review the types of ultralow flow LC technology, followed by a discussion of selected representative ultralow flow LC applications, focusing on the progress made in bioanalysis of amount-limited samples during the last 10 years. We also discuss several recently reported high-sensitivity applications utilizing flow rates up to 100 nL/min, which are below commonly used nanoLC flow rates. Finally, we discuss the path forward for future developments of ultralow flow LC.

## 1 | INTRODUCTION

Our world is completely in need of measurement sciences. Our knowledge about the air we breathe, the water we drink, the food we eat, and the blood we pump through our veins depends on chemical analysis and its performance and outcomes. While some analyses can be elegantly undertaken with small, portable sensors, other, more complex samples require more extensive analysis. Examples can be found when many analytes are to be measured, the analytes are prone to interferences with the sample matrix, and samples are very limited in size. A key technology for advanced measurements is MS. MS allows for highly selective and sensitive detection and quantitation of chemicals based on the measurement of their molecular mass-to-charge ratios. MS is routinely used for food analysis, environmental analysis, toxicology, doping analysis, diagnostics, and plenty more applications, where highly sensitive detection is needed. MS is also invaluable for omics analysis, that is, comprehensive mapping and measurement of a biological systemt’s metabolites (metabolomics), lipids (lipidomics), or proteins (proteomics). Tens of thousands of individual compounds can be measured, requiring that compounds are separated from each other prior to MS measurements, allowing the mass spectrometer to handle fewer compounds at a time, resulting in improved sensitivity and data output. Here, high-performance separation techniques come into play. The separation of nonvolatile molecules is typically performed using CE [1] or LC [2], which is by far the most commonly used separation technique coupled to MS. Therefore, LC has been a centerpiece and driving force in analytical chemistry for decades.

Most samples analyzed with LC-MS do not need special considerations regarding sample accessibility. For example, urine and blood are readily accessible in ample amounts. However, there is an increasing focus and need for bioanalysis and omics profiling of more amount-limited samples, for example, single cells, rare cancer stem cell populations, circulating tumor cells (CTCs), and extracellular vesicles including exosomes. In such cases, miniaturization of the LC system is often necessary, that is, downscaling the separation column id from the conventional 2.1 mm format to μm-scale ids. The downscaling allows for separated analytes to be less diluted when entering the detector [3], and lower LC flow rates allow for increased sensitivity and reduced signal suppression when using an ESI source for transferring compounds from the liquid phase to the gas phase for MS measurements.

**FIGURE 1:**
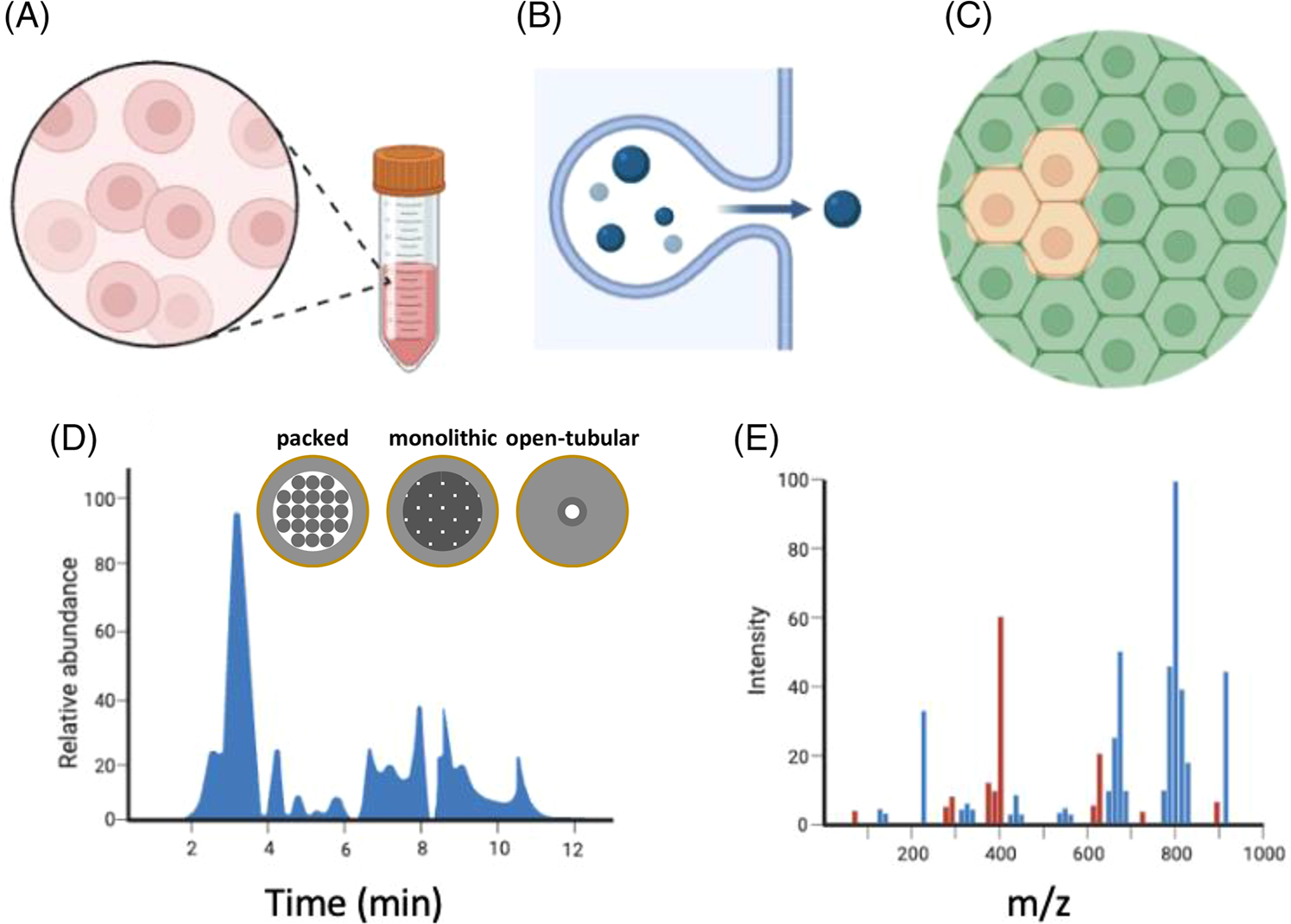
In this review, we discuss ULF LC and related systems for bioanalysis with a focus on limited samples. (A-C) Examples of limited samples: (A) single cells from cell cultures or biological fluids, (B) extracellular vesicles, and (C) single cells from tissues. (D) An illustration of a chromatogram and ULF LC column variants focused upon in this review, that is, particle-packed, monolithic, and open tubular. Related separation approaches, for example, CE (also operated below 100 nL/min) are not the focus of this review and have been reviewed elsewhere [1]. (E) MS is a typically used detection system.

In this review, we focus on cutting-edge LC miniaturization, namely ultralow flow (ULF) LC (Figure 1). We describe ULF LC-related technology and highlight selected bioanalytical applications of ULF LC from the last 10 years. ULF LC is typically associated with flow rates of 20–30 nL/min or less, but we also present some examples of related systems with flow rates up to 100 nL/min, as these systems are also highly useful for high sensitivity and amount-limited sample applications such as ULF LC. Last, we discuss the standing and potential of ULF LC and related approaches.

## 2 | ULTRALOW FLOW LIQUID CHROMATOGRAPHY

### 2.1 | Column formats

#### 2.1.1 | Particle-packed columns

By far, the most applied column variant in LC features capillaries packed with porous silica particles as a stationary phase, typically surface modified with a variant of an alkyl chain (e.g., C18). It has become quite commonplace to use sub-2 μm particles or solid core-shell variants for enhanced chromatographic performance [4, 5]. Particle-packed columns are routinely applied in many nanoLC variants, including ones with reduced flow rates, for example, in commercial systems with reproducible methods set to 100 nL/min. In addition, particle-packed columns have been employed for flow rates of approximately 20–50 nL/min using 20–30 μm id columns. Such columns are not widely commercially available and are typically prepared in-house [6, 7]. Although such columns enable high-performance analysis (see the Applications section below), particle-packed columns have limitations regarding how small beads can be used for packing and also how narrow capillaries can be used for column preparation, both with regard to high backpressure during manufacturing and use. In addition, frits, transfer lines, and other essential parts may contribute to the extra-column dead and dwell volumes of the system, which may also be extra challenging for further downscaling, in addition to several theoretical and practical challenges related to packing ultranarrow bore columns [8]. Therefore, it is worth-while to explore alternative or complementary variants, as described below.

#### 2.1.2 | Monolithic columns

Monolithic stationary phases for LC were introduced in the early 1990s, significantly promoted by Frantisek Svec [9–11], a key figure in the understanding, exploration, and advancement of separation science. Since then, monoliths have been an attractive alternative to particle-packed columns in the analysis of highly complex samples. In contrast to particle-packed columns, the continuous, porous structure does not require the use of frits, resulting in decreased sample losses on the surface of the frit and a simplified column preparation. Monolithic columns excel in ease of preparation, low backpressure, high permeability, the broad availability of surface chemistries, broad selectivity, noteworthy control over the structure and porosity of the stationary phase, and high separation efficiency [12, 13]. However, in the past, the monolithic columns suffered from inconsistent reproducibility of column preparation and performance that hampered their broader use outside the academic laboratories, while recent reports show that these issues are largely diminished [13, 14]. For ULF LC, high-efficiency ultra-narrow bore monolithic columns (≤20 μm id) play an important role since they significantly boost the sensitivity of nano ESI-MS.

**FIGURE 2:**
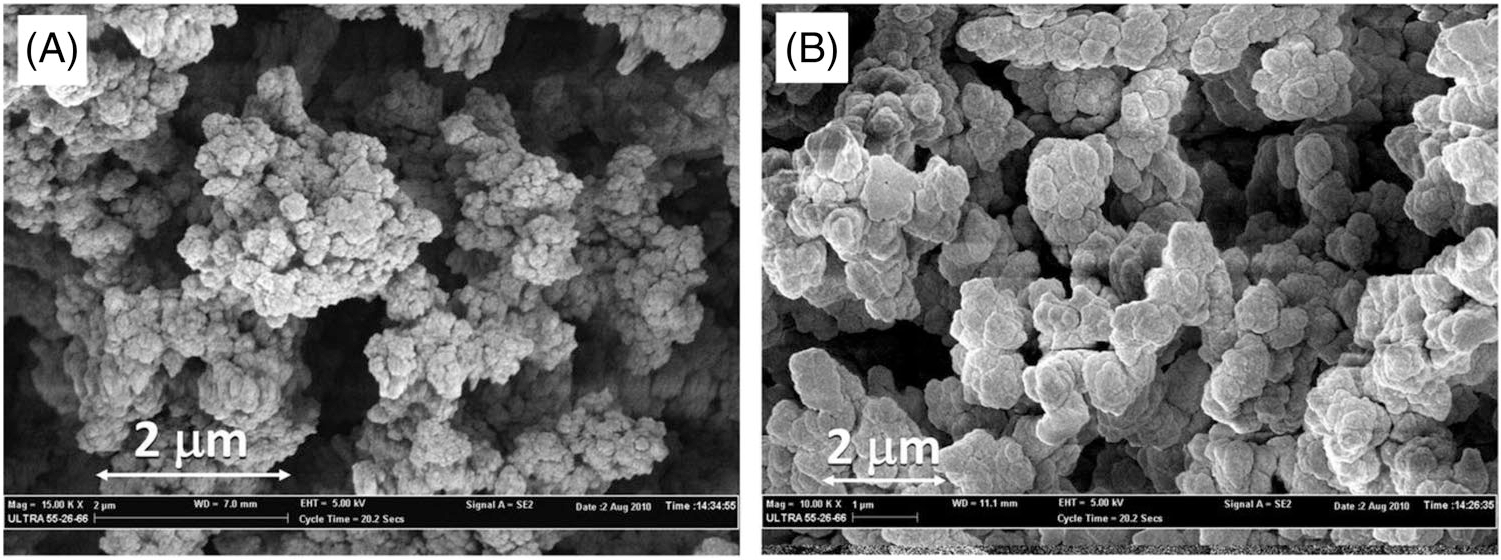
SEM images of porous hybrid organo-silica monolithic column prepared by UV-initiated photopolymerization using AIBN at two magnifications. Reprinted with permission from Wiley [22].

Most types of monolithic columns are prepared by *in-situ* polymerization initiated by temperature or light irradiation. The resulting separation medium of a monolithic column bed consists of a continuous, fully porous material made of both mesopores and macropores, resulting in high column permeability. Based on the chemicals used for monolithic column preparation, the monoliths can be classified into three main groups: organic [15, 16], inorganic [17, 18], and hybrid [19, 20]. Organic polymer-based monolithic columns are prepared from mixtures of monomers, crosslinkers, porogens (typically, a binary alcohol mixture), and a polymerization initiator. Methacrylates, acrylamides, and styrene monomers are the most widely reported in the scientific literature. On the other side, inorganic monolithic columns are typically silica-based, using tetramethoxysilane and tetraethyl orthosilicate as common reagents in the presence of PEG in a polymerization mixture. In addition are organic-silica hybrid monolithic columns [21]. Weed and colleagues prepared a porous hybrid organo-silica monolithic column in 100 μm id UV-transparent capillaries by a sol-gel transition and polymerization reaction [22] (Figure 2). The polymerization mixture consisted of 3-(methacryloyloxy)propyl trimethoxysilane in hydrochloric acid as a monomeric solution with various initiators and in the presence of toluene in the mixture, serving as a porogen. The authors explored various photoinitiators, namely azobisisobutyronitrile (AIBN) and Irgacure 819 (both irradiated at 365 nm), 2,2-dimethoxy-2-phenylacetophenone (irradiated at 254 nm), in addition to thermally initiated polymerization (at 80°C for 2 h) using AIBN. The prepared columns were evaluated by microflow HPLC. The best results in terms of efficiency were obtained using thermally initiated polymerization.

**FIGURE 3:**
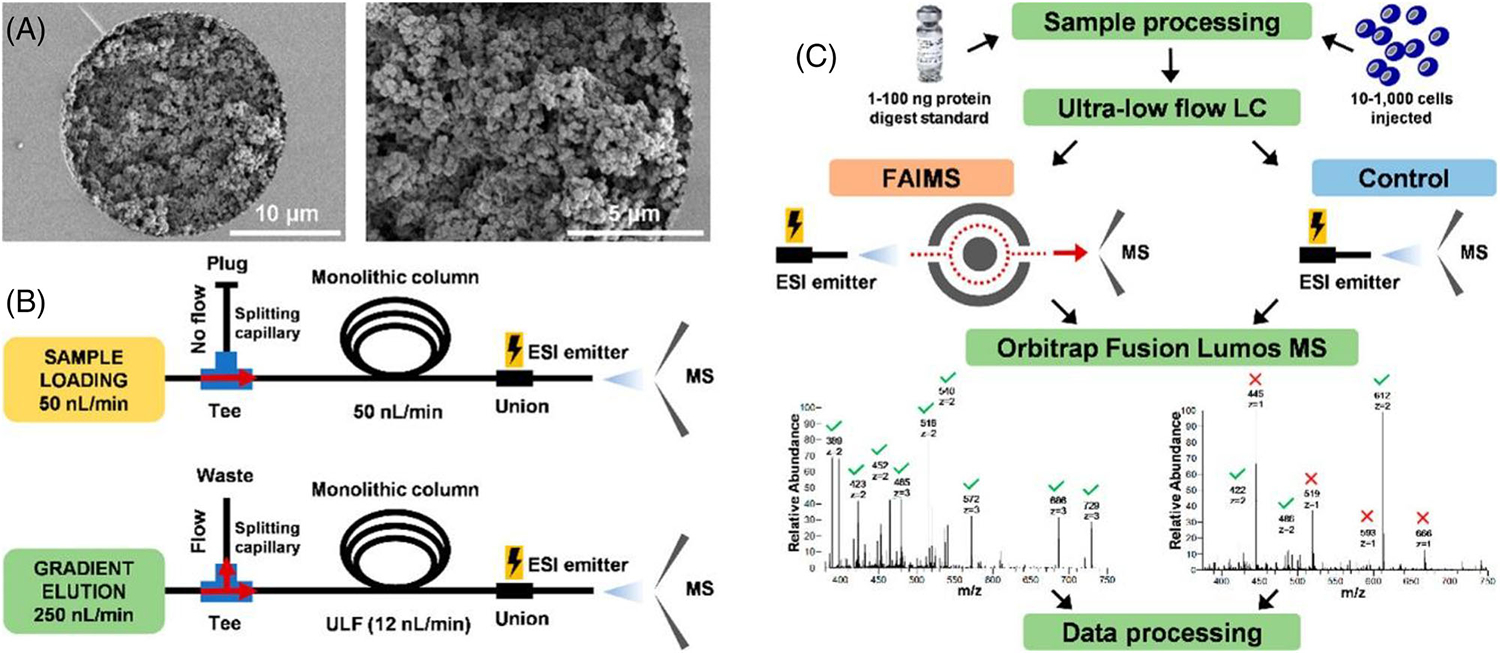
Overview of the ULF LC-MS experimental design and workflow developed by Gregus et al. (A) SEM images of the 20 μm id monolithic column. (B) Schematics of plumbing and configuration of the ULF LC–MS system. (C) The experimental workflows with and without the FAIMS Prointerface for in-depth proteomic analysis of model-limited samples. Reprinted with permission from [13]. (Copyright 2020, American Chemical Society).

In ULF LC, monolithic columns can also be utilized as trapping columns due to their low backpressure and high permeability, enabling quick sample loading and the absence of frits needed to keep the trapping media inside the capillary, allowing for easy back-flush and front-flush set-ups [23, 24]. However, monolithic columns can also serve standalone as analytical columns, as reported in a recent study [13] (Figure 3). Greguš et al. used a 20 *μ*m id (35 cm long) poly(styrene-divinylbenzene) (PS-DVB)-based monolithic column (copolymerized with C18 moieties) operated at 12 nL/min in combination with high-field asymmetric waveform ion mobility spectrometry (FAIMS) interface coupled to ultrasensitive MS in bottom-up proteomic analysis of model limited samples. The FAIMS interface significantly reduced the chemical noise, and the optimized method resulted in the identification of approximately 2400 protein groups and approximately 10,100 peptide groups from 1 ng HeLa digest (using a 1-h-long gradient). In addition, U937 myeloid leukemia cells were processed using an on-microsolid-phase extraction tip-based sample preparation workflow [25], and approximately 250, 1100, and 2200 protein groups were identified from analysis of only approximately 10, 100, and 1000 cells.

#### 2.1.3 | Open tubular columns

Open tubular (OT) LC columns [26, 27] can provide excellent sensitivity and separation efficiency and have been explored for decades, even for single-cell analysis (see Kennedy and Jorgensont’s work from 1989 [28]). However, the format has struggled to find a place in analytical science beyond niche studies and applications, perhaps due to its limited amount of stationary phase (i.e., low mass loadability), challenges with extra-column couplings and limited availability of pumps suited for pL-low nL/min flow rates. On the other hand, the last years have again demonstrated that the format can have considerable potential, perhaps most so regarding limited biological and clinical samples. The focus on small samples may be a solution to a common critique of the OT format, namely its limited sample capacity. While it may be hard to convince an analyst to use a technically challenging OT format over a 2.1 mm id column for ample amounts of a sample, it is difficult to envision that the increasing interest in small samples will be satisfied by “large” column formats. Therefore, OT LC may still see a future in the analysis of small samples, especially when coupled to a trapping column to alleviate the challenge with low loading capacity. OT columns can primarily be divided into three formats: bare open tubular (BOT; uncoated capillaries), porous layer open tubular (PLOT; relatively thick, porous wall coating), and wall-coated open tubular (WCOT; thin, non-porous layer wall coating) [29]. We discuss the latter two, as these are mostly associated with the analysis of biosamples.

##### Organic PLOT columns

PLOT columns with an organic polymer-based porous layer (e.g., PS-DVB polymer, ca. 0.5–1 μm stationary layer thickness) were explored by Karger and co-workers for bottom-up proteomics, allowing for ESI-MS-based analysis of a low number of cells [30, 31]. The column preparation protocol for the 10 μm id columns (several meters long) was found to be reproducible by Greibrokk and co-workers, who applied the columns for the separation of intact proteins [32], suggesting applicability for top-down proteomics as well. The same group applied the identical column format (up to 8 meters long), for mapping Wnt signal pathway-related proteins in pancreatic cancer cells [33]. In addition, PS-DVB PLOT columns can be applied for other applications and instrumentation than that associated with proteomics, for example, pesticide analysis coupled with ESI-MS [34]. Both Rogeberg et al. and Medina and co-workers observed that elevated temperatures could improve PLOT separations [32, 34]. The name “Porous layer open tubular” may be misleading, as for some recipes, the presence of pores has not been established [35]. Therefore, “polymer layer open tubular” may be a more suited name for organic PLOT columns.

##### Inorganic PLOT columns

OT LC can also be prepared using silica support layers. While the organic PLOT variants seem to be more associated with peptides and proteins, the silica variants are also well-suited for smaller molecules. One of the major developments in this direction within the past decade is the collaborative work of Hara and Desmet [36–38]. In their initial study, they coated 5 μm id capillaries with mesoporous silica layers with a thickness of up to 550 nm. For 60 cm long columns, the efficiencies could be as high as *N* = 150 000, depending on the layer thickness, and up to 600 000 for ca. 2-m-long columns [37]. In follow-up publications, the hydrothermal treatment PLOT column preparation process was modified, to reduce the size of mesopores [38] and to increase the stationary phase hydrophobicity [36]. These columns were operated at a linear velocity of approximately 0.6–1.5 mm/s roughly corresponding to 0.7–1.8 nL/min flow rate.

##### WCOT columns

**FIGURE 4:**
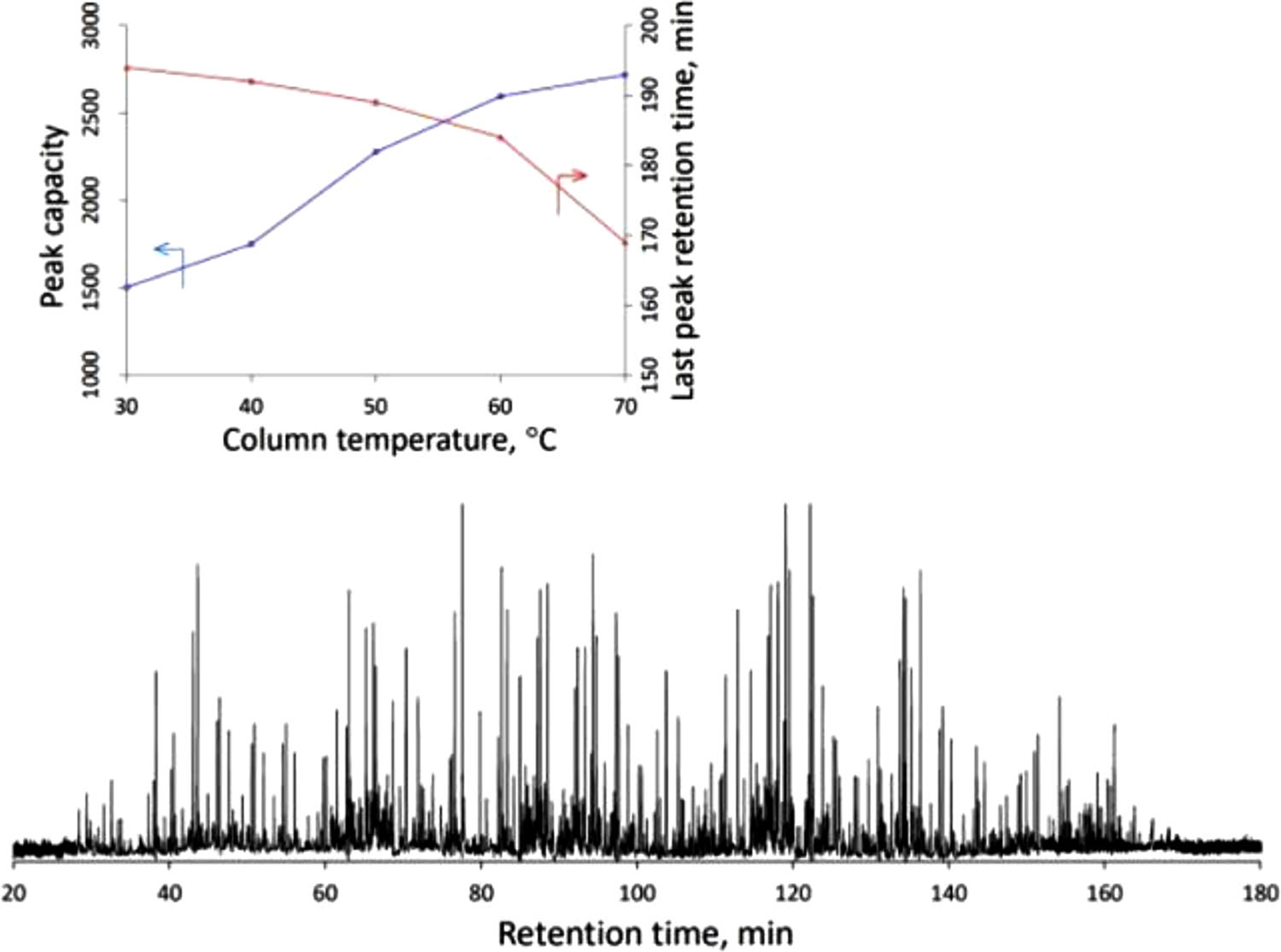
Separation of protein digests using a 2 μm id × 160 cm long OT column developed by Liu and co-workers [39]. Top: The effect of temperature on peak capacity and the last peak retention time. Bottom: A chromatogram with a peak capacity of 2720 within 143 min (70°C separation temperature). Modified with permission (Copyright 2021, American Chemical Society).

Liu and co-workers have pushed the limits of id even further, demonstrating excellent chromatography of peptide samples using 2 μm id WCOT columns (non-porous layers) [39]. Using a 160 cm long column at elevated temperatures, peak capacities of close to 3000 were obtained; such outstanding numbers demonstrate the clear potential of using ultra-narrow columns for complex samples (Figure 4). A potential weakness of WCOT columns can be a limited sample capacity but Lie et al. addressed this issue through the use of narrow columns (high surface-to-volume ratio) with dense nonporous layers [40]. The same group has also performed very rapid separations with WCOT columns (baseline separation of six amino acids in less than 700 ms) [41].

#### 2.1.4 | Alternative variants: Nanoparticles and OT format

**FIGURE 5:**
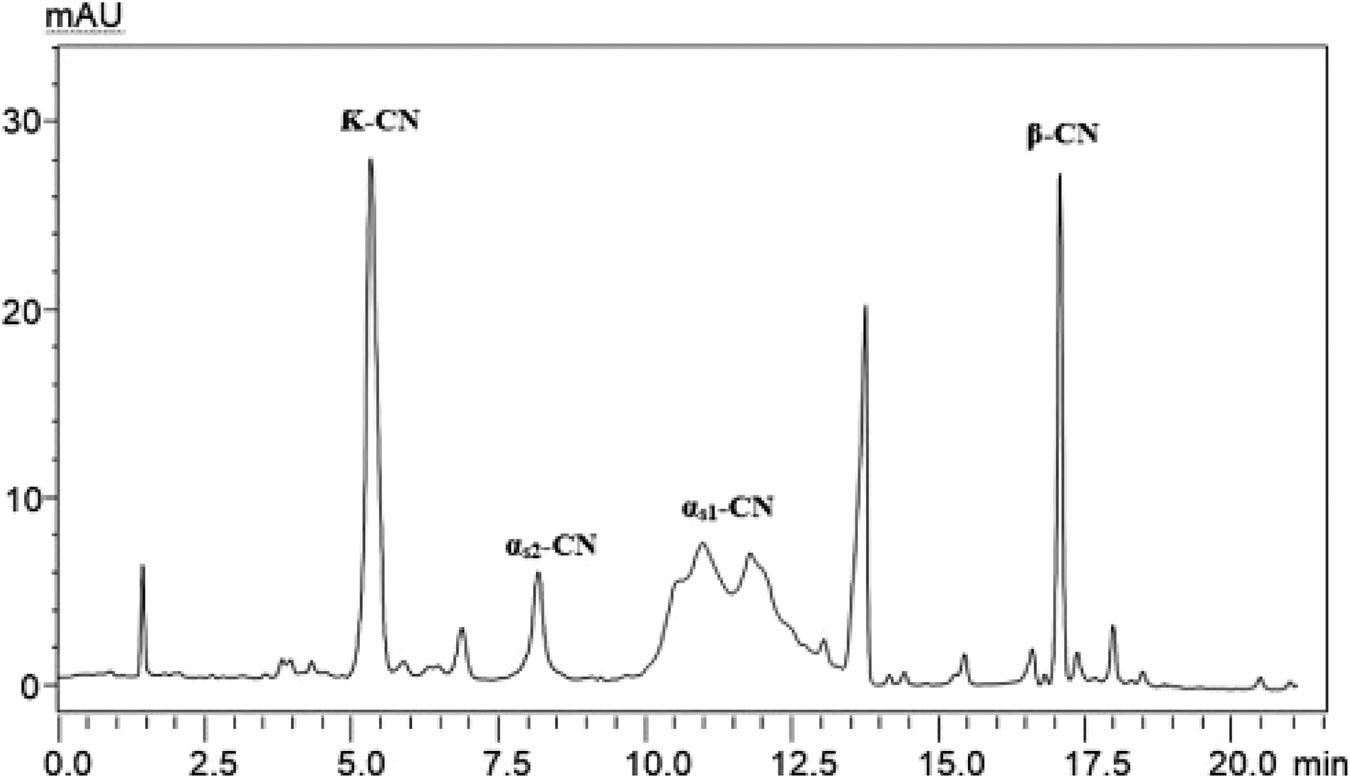
Separation of casein protein variants, including α_s1_-CN, α_s2_-CN, β-CN, and κ-CN, using an OT nanoLC column with polylysine grafted on graphene oxide stationary phase [42]. Reprinted with permission from Elsevier Publishing.

As an example of an alternative OT format, Seker and co-workers prepared narrow OT columns (i.e., 20 μm id) that utilized graphene oxide nanoparticles [42]. A poly-L-lysine was grafted on vinylized graphene oxide, creating a stationary phase that featured both electrostatic and hydrophobic interactions. The column suitability was demonstrated through the separation of casein protein variants, as shown in Figure 5. The column-to-column reproducibility was satisfactory, with ~3% RSD retention time variation, tested with five columns.

### 2.2 | Coupling with online sample preparation, trapping, and delivery

In contrast to conventional LC systems, sample injection onto miniaturized systems is not straightforward, as injection volumes above pL/low nL volumes may reduce the overall performance of the chromatographic system. Here, it is important to note that the volume of a 10 μm id capillary is in order of nL (~8 nL for a 10 cm long capillary), and typical injection volumes in order of the microliters will simply not work. Therefore, in some proof-of-concept studies, the simplest approach (i.e., sample splitting) is used. In this case, the sample is injected using a splitting tee connected to a separation column and a splitting capillary. Only a small portion of the sample (usually < 1% of the total sample volume) is directly injected as a short sample plug into the analytical column; the rest is diverted through the splitting capillary to the waste. Consequently, this type of sample injection comes with the expense of irreversible sample loss [37, 43].

Therefore, in most cases, online SPE is used for enriching and focusing a sample prior to separation, as even 1 μL samples can overload an ultranarrow column and require a significant loading time, resulting in peak broadening and/or extended analysis times. SPE columns may need to have lower retention of analytes compared to the analytical column to ensure a band refocusing on the analytical column upon elution of analytes from the trapping column. Medina et al. described that “straight-flush” trapping and elution can result in the improved separation performance compared to the “back-flush” mode [34]. SPE columns can be filled with a solid phase particulate, monolithic media, or feature an OT format (e.g., multichannel geometry for increased capacity, see [44]). SPE (trapping) columns combined with downstream ULF analytical columns are typically made in ca. 50 μm-scale id, allowing for fast sample loading and, ideally, a highly reproducible manufacturing/synthesis. In fact, we believe that the difficulties in producing high-quality SPEs and connections for ultra-narrow columns are an underestimated bottleneck in the advancement of low-flow systems.

**FIGURE 6:**
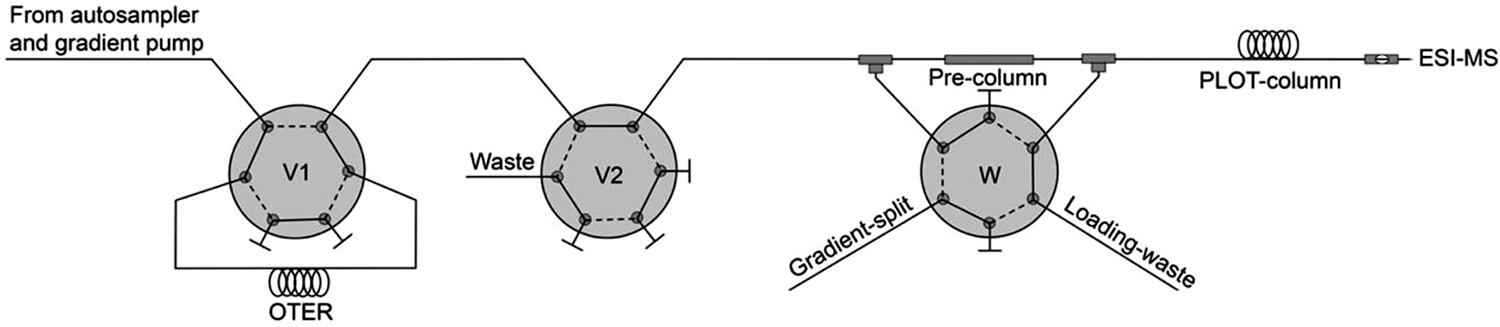
Online open tubular enzyme reactor (OTER) for cleaving proteins into peptides, followed by PLOT separation of the peptides prior to mass spectrometric analysis. Samples were loaded onto the OTER (~1.2 μL) at a flow rate of 0.5 μL/min. The OTER was placed inside a column oven at 37°C. Generated peptides were eluted from the OTER onto a 50 μm id × 4 cm butyl-methacrylate monolithic precolumn, to trap and desalt the peptides. The flow was split to 40 nL/min by switching valve W, and peptides were separated using a linear gradient on a 10 μm id × 500 cm long PS-DVB PLOT column. Figure from [45]. Reprinted with permission under a CC-BY license.

Even though SPE systems can pose challenges in production and installation, some examples demonstrate the great potential of the approach, also combined with other sample preparation steps. For the analysis of HCT15 colon cancer cells, Hustoft et al. placed a combination of SPE monolithic trap column coupled to the PLOT LC column downstream to an OT enzyme reactor (featuring both trypsin and Lys-C) for online digestion of proteins prior to enrichment and separation of resulting peptides (Figure 6) [45]. Even with this multistep system, the resulting chromatography was of high quality and with low carry-over. As we discuss in the following applications, online sample handling and tailored sample preparation steps are commonly featured with ULF LC systems.

Another alternative for sample introduction is based on the Stage Tip technology, and was later adopted by the Evosep Company [23, 25, 46]. This platform uses disposable trap columns (i.e., Stage Tips) called Evotips, where samples are loaded and desalted offline. The autosampler then picks up the tip with trapped peptides and places it online with a low-pressure pump system for gradient elution. The eluent (eluted peptides in a gradient that are “diluted” with the water-organic solvent-based gradient) is stored in a long capillary loop and later pushed with a high-pressure pump through the analytical column at a flow rate as low as 100 nL/min with a water-based solvent [47].

## 3 | SELECTED ULTRALOW FLOW LIQUID CHROMATOGRAPHY APPLICATIONS TO BIOLOGICAL SAMPLES

### 3.1 | Lipids, extracellular vesicles, and exosomes

**FIGURE 7:**
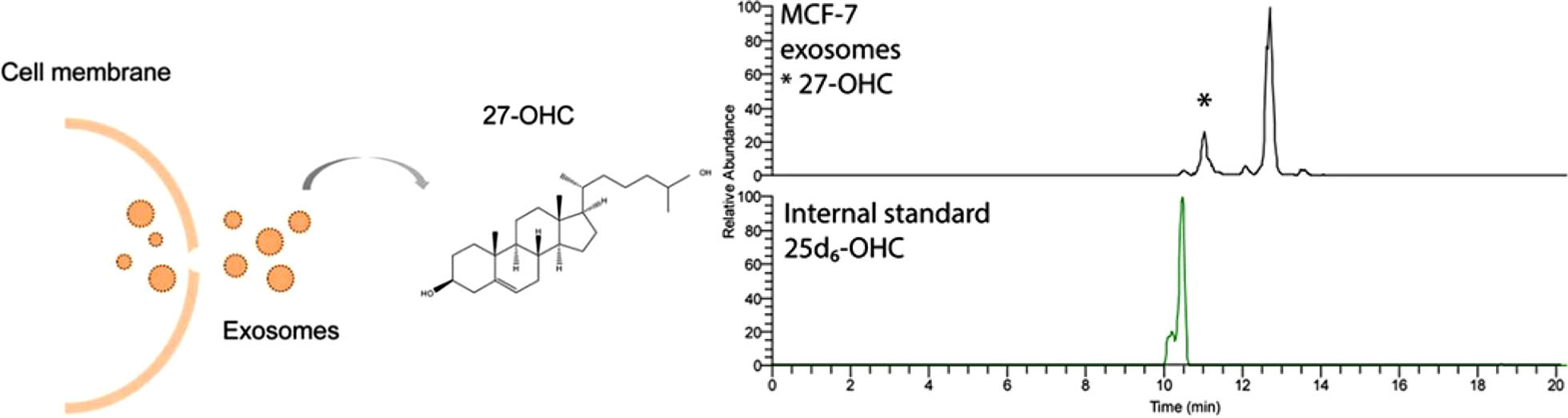
Exosomes are extracellular vesicles released by cells. OT LC-MS was suited for separating and detecting 27-OHC lipids in exosomes from breast estrogen receptor-positive cancer cells. Reprinted with permission through CC-BY license.

Silica-based OT columns can be surprisingly versatile. Using silica layer-based 10 μm id columns (3 m long) functionalized with octadecyl groups and a PS-DVB-based enrichment column, Vehus et al. demonstrated that the column could be employed for endogenous metabolites, peptides, and intact proteins, operated at 25–50 nL/min [24]. Regarding metabolites, the analytes were hydroxycholesterols (also called oxysterols), which are lipids associated with many developmental and signaling processes, but also linked to proliferation and metastasis in cancer (for example, 27-hydroxycholesterol = 27-OHC [48]). Oxysterols can be difficult to analyze, as they require a derivatization step to obtain adequate ESI sensitivity. In addition, important oxysterols, such as side-chain hydroxylated variants, are present as isomers that require a separation step. Moreover, when studied in limited samples, detection limits become an increasing issue. One such type of limited sample is exosomes, small extracellular vesicles released by cells that play a role in intercellular communication by transferring proteins, lipids, and genetic material between cells [49, 50]. Vehus et al. demonstrated that silica-based OT columns combined with an Orbitrap Q Exactive MS instrument (which enabled low attogram-level detection limits) could separate and detect 27-OHC in exosomes from MCF-7 breast cancer cells (Figure 7).

### 3.2 | Proteomic profiling of rare cells in whole blood

**FIGURE 8:**
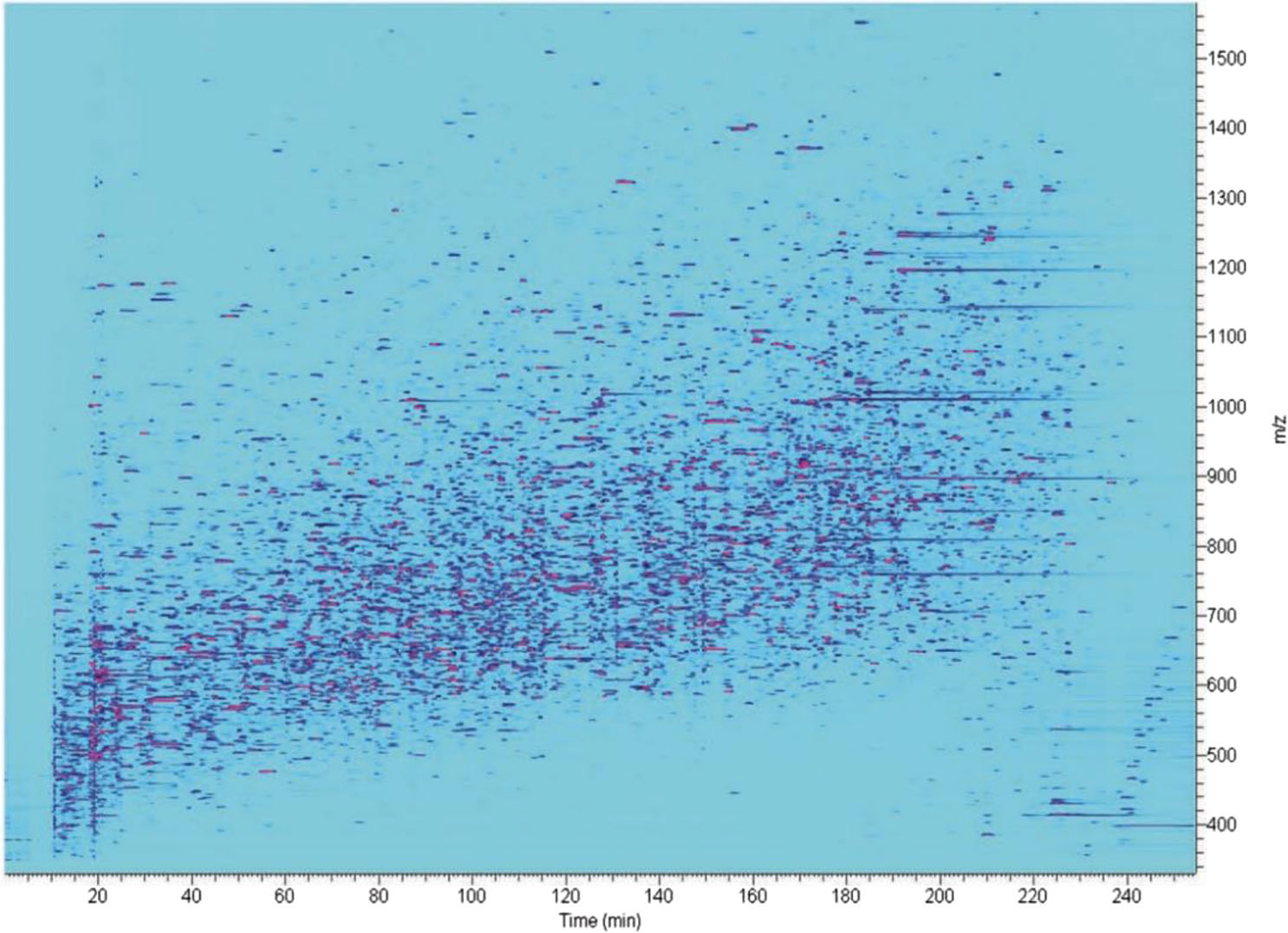
The ion density map shows the analysis of 100–200 MCF-7 cells isolated from 1 mL blood sample using a PLOT column (240 min separation) coupled to ultrasensitive MS. Figure from [23]. Reprinted with permission through CC-BY license.

The isolation of rare cells from biological matrices, such as blood, can allow for diagnostic opportunities and the collection of cells that can be utilized for bioengineering purposes. However, the selective extraction of specific cells, for example, CTCs, can be challenging in complex matrices. Targeted cells, a limited subcomponent of the total sample (as in the case of the previous examples), can result in sensitivity challenges. Ivanov and co-workers established a platform for isolating and proteomic profiling of endothelial progenitor, hematopoietic, and CTC cells from whole blood demonstrated with MCF-7 (EpCAM+) and CD133^+^ cells [23]. Microfluidic magnetophoretic isolation of target cells (1000–2000 cells/mL) was undertaken, followed by focused acoustics-assisted sample preparation. The cells were subsequently analyzed using polymer-based PLOT columns (4 m long, 10 μm id with ~1 μm stationary layer thickness). The zeptomole detection sensitivity of the system allowed for the identification of ca. 4000 proteins from only 100–200 cells per analysis. For the separations, the authors applied a 4-h long gradient at a flow rate of ~20 nL/min. See Figure 8 for a retention time/mass-to-charge ion density map of separation using the above-mentioned samples and conditions.

### 3.3 | Gold nanoparticle-modified OT LC system, combined with online pretreatment of living cells

**FIGURE 9:**
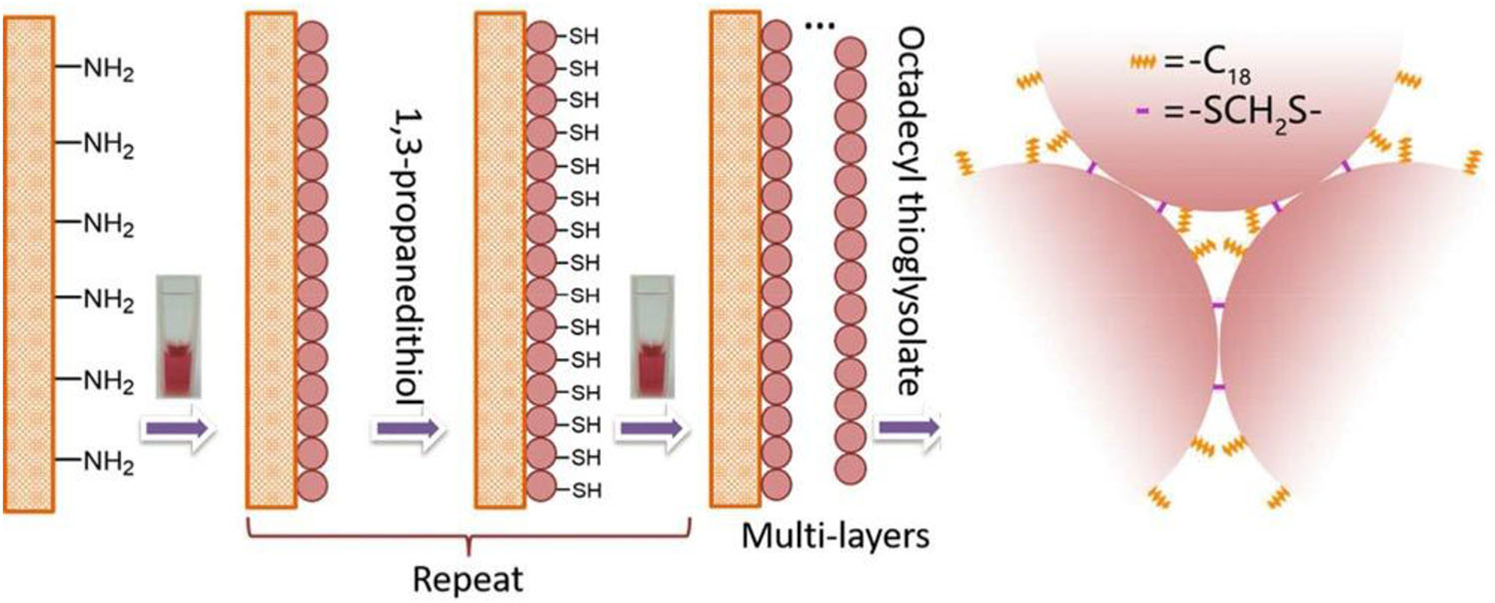
Preparation of an OT LC column modified by five-layer gold nanoparticles that were linked with C_18_ for reversed-phase separations. Figure from [51]. Reprinted with permission from Wiley Publishing.

Shao and Zhang developed an OT LC column (2 m × 20 μm id) that was modified by five-layer gold nanoparticles that were linked with C_18_ for reversed-phase separations of peptides (GNPs@C_18_) (see Figure 9) [51]. The column was used to separate peptides for bottom-up proteomics, of 80 living HepG2 liver cancer cells, prior to mass spectrometric analysis. Approximately 650 proteins were identified in triplicate runs. Interestingly, a sample preparation or pretreatment was placed upstream of the separation and detection system for on-loop cell digestion, followed by peptide trapping. The GNPs@C_18_ system had a 30–100 fmol loadability, depending on the control compounds investigated. One of these test compounds was insulin, which could be an important analyte to pursue further for other limited samples, for example, Langerhans islets.

### 3.4 | Single cell-level omics

**FIGURE 10:**
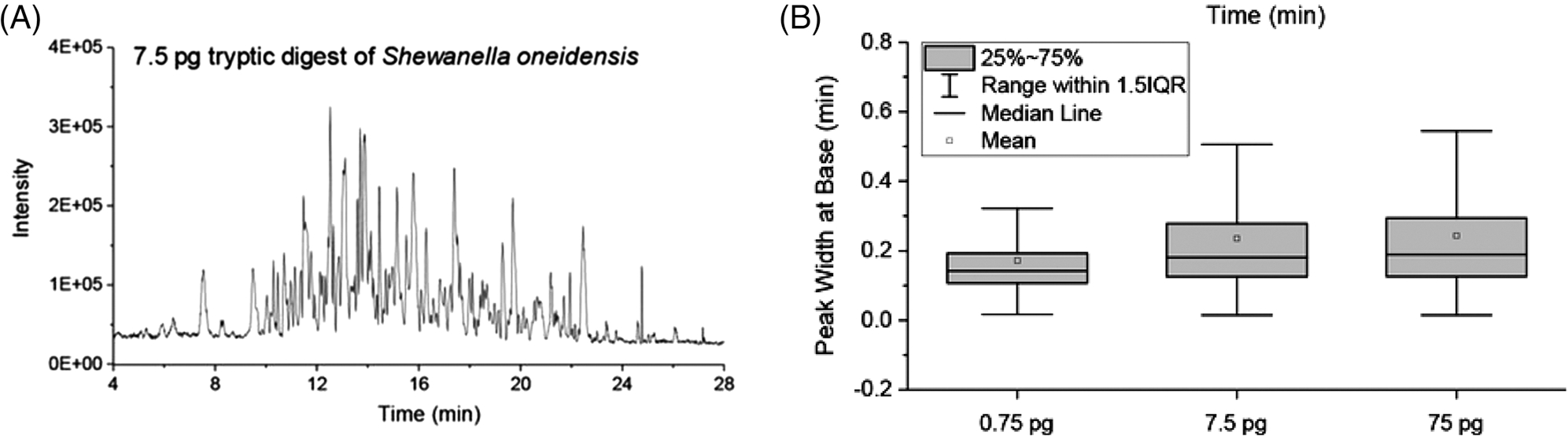
PicoLC-MS, with sub-nL/min flow rates, enables high-resolution separations of limited proteomics samples. Figure from [43]. Reprinted with permission. Copyright 2023 American Chemical Society.

Single-cell analysis can help us understand the composition, heterogeneity, and cell-to-cell interactions within a complex sample, such as an organ tissue or a tumor. A typical mammalian single cell consists of approximately 100–500 pg protein. Such a limited sample places substantial considerations regarding the various proteomic preparation steps, including cell lysis, labeling, enzymatic digestion, and extraction. In addition, the chromatography step prior to detection must be tailored for very small sample amounts while providing high-resolution separations of analytes of a highly complex sample. OT LC has been applied for single cell-level samples by Liu and colleagues [43]. Here, the authors employed a 2-μm id OT column in combination with MS detection. With this “picoLC-MS” system (~800 pL/min LC flow rates), the authors could identify approximately 1000 proteins reliably using only 75 pg of tryptic peptides. The narrow OT column featured a C18 stationary phase. Serially diluted tryptic peptide samples from *Shewanella oneidensis* bacteria were analyzed using a 30-min LC gradient, see Figure 10. Prior to the picoLC-MS analysis, the authors employed a robotically addressed chip-based nanodroplet sample preparation platform (nanoPOTS = Nanodroplet processing in one pot for trace samples) that is tailored for limited samples.

Recently, Kelly and co-workers published a method for multiplexed single-cell analysis using ULF LC conditions [52]. Using a combination of isobaric and isotopic labeling (tandem mass tag and stable isotope labeling by amino acids in cell culture), up to 28 single cells could be analyzed in a single LC-MS run. The columns used were a home-particle packed 100 μm id SPE column for desalting, and a 30 cm long × 30 μm id LC analytical column, both packed with 1.9 μm silica particles with a C18 stationary phase. A flow rate of ~30 nL/min was used with a 60-min solvent gradient (145-min total LC cycle time) for separating peptides of digested cells. The system allowed coverage of ~800 proteins per cell and was able to distinguish between different cell types. The work clearly points to the throughput potential of ULF LC for single-cell analysis when combined with effective sample preparation and high-resolution MS.

### 3.5 | Spatial single-cell mass spectrometry for analyzing complex tissues

As we discussed, the analysis of single cells from cultures is well within reach using ULF LC-MS. Mann and co-workers have recently posted a preprint describing the use of a 100 nL/min flow rate LC system (Evosep) for the analysis of single cells collected using laser microdissection, enabling spatial analysis at a single-cell level [53]. Using multiplexing, the authors could map approximately 1700 proteins per cell, allowing for the exploration of spatial characteristics of hepatocyte subsets in the mammalian liver. The instrumentation applied was commercially available, and the authors used 150 μm id particle-packed columns (relatively wide compared to many other ULF LC systems described in this review).

## 4 | CONCLUDING REMARKS

The introduction and applications that we have presented above can illuminate several points. ULF LC conditions are indeed suited for different types of biological samples and analytes, limited samples, and even high throughput applications. ULF LC can provide outstanding performance in respect to the resulting selectivity and sensitivity, typically obtained using ≤1 h-scale gradients, but ULF LC can also be applied for very rapid separations of biomolecules [40]. By 2023, ULF LC and related approaches proved themselves as powerful tools for generating highly informative molecular profiling results to ultimately improved our understanding of biology and health. Particle packed columns, monoliths, and OT variants all show considerable promise and are difficult to rank which shows the most promise at this time. In addition, we predict that the pillar array format will become a strong tool for ULC LC in the figure, as we see that the format is already becoming established, for example, in single cell-scale proteomics [54].

As we showed in the representative examples above, high performance separation conducted using pL/min—low nL/min flow rates can provide striking results when interfaced with MS. However, the lack of availability of the LC instrumentation to deliver such low flow rates without the penalty in sample loss or decreased throughput currently leads to the effective and more commonly used applications of state-of-the-art systems delivering higher flow rates (e.g., ≤100 nL/min), which can be powerful in achieving a reasonable balance between the throughput and profiling sensitivity in analysis of amount-limited samples. For example, Mechtler and co-workers recently demonstrated an approximately 100 runs/per day throughput (with ca. 1700 protein groups identified per single cell), using a 50 μm id packed column, operated at 100 nL/min [55]. A key component in the approach was optimizing method’s duty cycle to allocate as much time as possible to MS data acquisition while minimizing the time allocation for column equilibrations, washes, etc. A key point can be that the LC column, stationary phase, flow rate, and other separation conditions are only part of the whole story. Optimized performance in each and every stage of the process, starting from sample procurement, sample preparation, and following with sample transfer with minimal losses, separation, interfacing to MS, MS data acquisition, data analysis and interpretation, as well as robustness, reproducibility, and throughput of every stage, individually and in combination, is the key in the successful execution of limited sample analyses. In the current era of the increased research and method development activity in the field of single-cell omics analysis, we are hopeful that more emphasis will be placed on the development of specialized ULF LC platforms to enable splitless delivery of the ULF by their pumps, containing low dead and dwell volumes to minimize gradient delay and distortion, injection of pL/nL-scale samples from various sample vessel geometries, and minimized sample losses.

Although this review has placed a significant emphasis on the coupling of ULF LC and MS, ULF LC is not necessarily dependent on MS for success and relevance in some applications. As microfluidic chip-based systems and other miniaturized analytical platforms are becoming more attractive research tools (take organ-on-a-chip platforms as an example [56]), there literally might not be room for a mass spectrometer in an integrated device. However, it is a safe bet to assume that miniaturization will continue to be a critical direction, as one of the analytical research advancements, and the need for high-resolution separations and high sensitivity molecular detection, profiling, and characterization will be in substantial demand. Therefore, we suggest that ULF LC techniques coupled with smaller and simpler detection systems (e.g., LIF detection) in addition to MS-based analyses will continue to be explored by multidisciplinary research laboratories.

## Data Availability

Data sharing is not applicable to this article as no new data were created or analyzed in this study.

## Article Related Abbreviations:

27-OHC: 27-hydroxycholesterol
AIBN: azobisisobutyronitrile
BOT: bare open tubular
CTCs: circulating tumor cells
FAIMS: high-field asymmetric waveform ion mobility spectrometry
GNPs@C18: gold nanoparticles linked with C18
OT: open tubular
OTER: online open tubular enzyme reactor
PLOT: porous layer open tubular
PS-DVB: poly(styrene-divinylbenzene)
SILAC: stable isotope labeling by amino acids in cell culture
ULF: ultralow flow
WCOT: wall-coated open tubular
